# Supplementary material for: Chondrocyte Thrombomodulin Protects against Osteoarthritis
Source: Int J Mol Sci. 2023 May 30;24(11):9522. doi: 10.3390/ijms24119522 (PMC10253941; doi:10.3390/ijms24119522)

Supplementary Figure S1. Immunohistochemical (IHC) staining was performed on articular cartilage sections obtained from both normal human subjects and patients with OA (osteoarthritis). Purchased sections were performed to IHC staining to evaluate the TM level of chondrocytes. Scar bar: 100 μm.

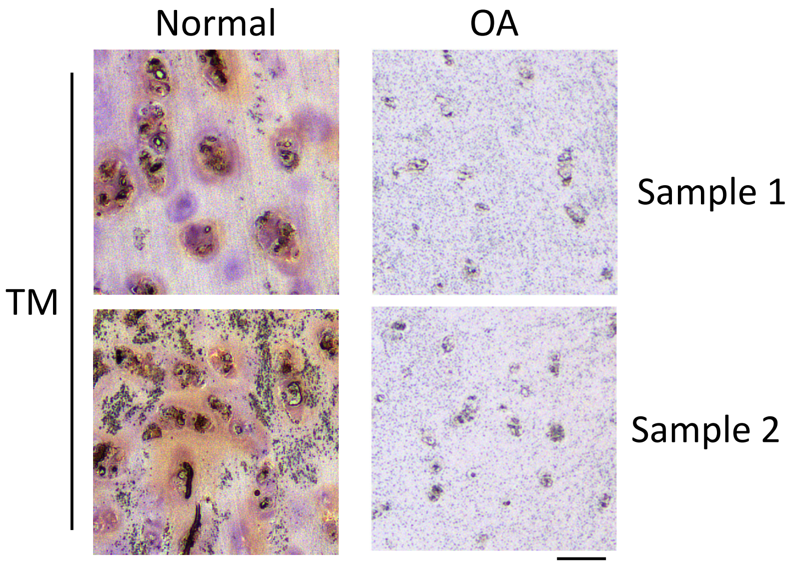

Supplement: Supplementary file 1 [file ijms-24-09522-s001.zip › ijms-2406880-supplementary.pdf]
